# Use of ECG and Other Simple Non-Invasive Tools to Assess Pulmonary Hypertension

**DOI:** 10.1371/journal.pone.0168706

**Published:** 2016-12-28

**Authors:** Gabor Kovacs, Alexander Avian, Vasile Foris, Maria Tscherner, Xhylsime Kqiku, Philipp Douschan, Gerhard Bachmaier, Andrea Olschewski, Marco Matucci-Cerinic, Horst Olschewski

**Affiliations:** 1 Medical University of Graz, Department of Internal Medicine, Division of Pulmonology, Graz, Austria; 2 Ludwig Boltzmann Institute for Lung Vascular Research, Graz, Austria; 3 Medical University of Graz, Institute for Medical Informatics, Statistics and Documentation, Graz, Austria; 4 Medical University of Graz, Institute for Physiology, Graz, Austria; 5 Department of Biomedicine, Division of Rheumatology, AOUC and Department of Experimental and Clinical Medicine, University of Florence, Florence, Italy; JAPAN

## Abstract

**Background:**

There is a broad consensus that pulmonary hypertension (PH) is to be diagnosed by right heart catheterization (RHC) and that the most important non-invasive tool is echocardiography. However, the role of simple non-invasive tools in the work-up of PH is not clearly defined. We hypothesized that the use of simple non-invasive techniques may help to guide important decisions in the diagnostics of pulmonary hypertension.

**Objectives:**

We aimed to develop an algorithm with the use of simple, non-invasive tools in order to identify patients with very high or very low likelihood of PH.

**Methods:**

We retrospectively analyzed all consecutive patients undergoing RHC between 2005 and 2010 in our center and performed logistic regression of simple non-invasive parameters regarding detection and exclusion of PH and derived a two-step algorithm. In a prospective study we evaluated this algorithm between 2011 and 2013.

**Results:**

The retrospective cohort consisted of n = 394 patients of which 49% presented with PH. Right axis deviation in the ECG was present in 90/394 patients and had a positive predictive value (PPV) of 93% for PH. The combination of non-right axis deviation, N-terminal pro brain natriuretic peptide (NT-proBNP)<333pg/ml, arterial oxygen saturation (SO_2_)≥95.5% and WHO functional class I-II was present in 69/394 patients and excluded PH with a negative predictive value (NPV) of 96%. The prospective study confirmed these results in a cohort of n = 168 patients (PPV:92%, NPV:97%). Taken together, simple non-invasive tools allowed a prediction regarding the presence or absence of PH in 42% of patients with suspected PH.

**Conclusion:**

ECG, NT-proBNP, SO_2_ and WHO functional class may predict the presence or absence of PH in almost half of the patients with suspected PH, suggesting an important role for these variables in the work-up of patients at risk for PH.

**Clinical Trial Registration:**

NCT01607502

## Introduction

Pulmonary hypertension (PH) is a severe hemodynamic condition of the pulmonary circulation eventually leading to right heart failure and death [1]. Independent of its etiology, development of PH is associated with worse prognosis of the underlying disease [2–4]. PH is diagnosed by right heart catheterization [1], while Doppler echocardiography, with all its limitations [5], is widely accepted as the most specific non-invasive screening tool. Other simple, widely available, non-invasive tools such as ECG, chest X-ray, pulmonary function tests (PFT), blood gas analysis (BGA) and laboratory tests are recommended to be performed during the diagnostic process [1], but their specificity and sensitivity is generally considered to be low, often restricting these methods to delineate co-morbid conditions, or, in the best case, to support the suspicion of a pulmonary vascular disease. A recent study in systemic sclerosis patients, however, revealed that a combination of simple non-invasive markers performed better than echocardiography alone and recommended a complex algorithm that helps to avoid some echocardiographic and right heart catheter investigations [6]. As limitations, the study included only systemic sclerosis patients with a low diffusing capacity for CO (DLCO) and the results may not be relevant for other populations than systemic sclerosis patients. In our study, we derived a two-step algorithm from a retrospective evaluation and we applied this simple algorithm in a prospective cohort of patients. The algorithm is based on a combination of simple, investigator-independent, non-invasive examinations that may be available in almost every outpatient clinic and may help to guide further diagnostic decisions in the work-up of pulmonary hypertension.

## Patients and Methods

The study consisted of a retrospective part, in which a combination of parameters was identified by logistic regression and based on which an algorithm was developed, and a validation part, in which the proposed algorithm was tested in a prospective manner. Between 2005 and 2010, patients of the Medical University of Graz undergoing right heart catheterization due to suspected pulmonary hypertension were included into the retrospective part of the study. In the prospective part, patients undergoing right heart catheterization (RHC) between 2011 and 2013 were evaluated. All patients gave written informed consent. All patients had either unexplained dyspnea or had an established risk factor for PAH (e.g. systemic sclerosis) and the diagnostic work-up followed the recommendations of current international PH guidelines [7,8]. The study has been approved by the local ethics review board. In all patients, right heart catheterization and a routine non-invasive assessment [1] including physical examination, history, ECG, blood gas analysis, pulmonary function tests, laboratory tests and six-minute walk test was performed. As the goal of the study was the development of a simple algorithm, based on the routine work-up of PH patients, the following simple parameters were evaluated: ECG: the presence of right axis deviation (RAD) characterized by an electrical heart axis greater than +90° (the amplitude of the S wave bigger than the amplitude of the R wave in lead I); blood gas analysis: arterial partial pressure of oxygen (pO_2_), arterial partial pressure of carbon dioxide (pCO_2_), arterial oxygen saturation (SO_2_); pulmonary function tests: forced expiratory volume in the first second (FEV_1_), forced vital capacity (FVC), diffusion capacity for carbon monoxide (DLCO); laboratory tests: N-terminal pro brain natriuretic peptide (NT-proBNP), uric acid; six-minute walk distance, Borg dyspnea score at the start and the end of the six-minute walk test; WHO functional class.

ECG was reviewed for RAD by two independent physicians; unclear cases were decided by consensus. Blood gas analysis of arterialized ear lobe capillary blood was performed with an ABL 800 Flex (Radiometer; Copenhagen, Denmark) blood gas analyzer. Pulmonary function test was performed with a Jaeger MS PFT Analyzer, NT-proBNP and uric acid levels were determined by commercially available kits. Six-minute walk tests were performed according to the recommendations of the American Thoracic Society [9]. RHC examinations were performed with a 7F quadruple-lumen, balloon-tipped, flow-directed Swan-Ganz catheter (Baxter) in the supine position using the transjugular approach. The reference point was set at the level of the anterior axillary line [10].

The study was approved by the Ethics Committee of the Medical University of Graz (NR: 23–408 ex 10/11). Participants provided their written informed consent to participate in this study. The ethics committee approved this consent procedure.

### Statistics

Data are presented as mean and standard deviation or median and interquartile range for continuous data and absolute and relative frequency for categorical data, respectively. To identify patients with a high risk for PH and patients with a very low risk for PH in the retrospective analysis, a two-step algorithm was developed. In the first step, patients with right axis deviation (electrical axis of the heart greater than +90°) were selected as high risk patients. In the second step, in the remaining patient group, possible predictors (arterial SO_2_, pO_2_, pCO_2_, DLCO, FEV_1_, FVC, Borg dyspnea score at the end of the six minute walk test, NT-proBNP, uric acid, 6 Minute Walking distance and WHO functional class) for exclusion of PH were analyzed. In the univariate logistic regression analysis the predictors’ ability for discriminating patients with and without PH (mean pulmonary arterial pressure (mPAP) ≥ 25mmHg vs. mPAP < 25mmHg) was analyzed. Univariate significant variables were checked for multicollinearity. Multicollinearity was verified by correlation matrices: a correlation >0.4 was used as the cutoff for multicollinearity. After excluding multicollinearity, remaining variables were selected for multivariate logistic regression. Variables in the final model were selected with a forward stepwise procedure. The decision to include variables was based on a likelihood-ratio test. To calculate the best cut off score of each variable we used the Youden-Index. Therefore the value of a variable which maximizes sensitivity and specificity was calculated by using ROC analysis. The result was evaluated in the prospective cohort. Sensitivity, specificity, negative predictive value and positive predictive value were calculated for both steps and for the final model. A p-value <0.05 was considered significant. Statistical analysis was performed with IBM SPSS Statistics (Release 20.0.0. 2011. Chicago (IL), USA: SPSS Inc., an IBM Company) software.

## Results

**Fig 1:**
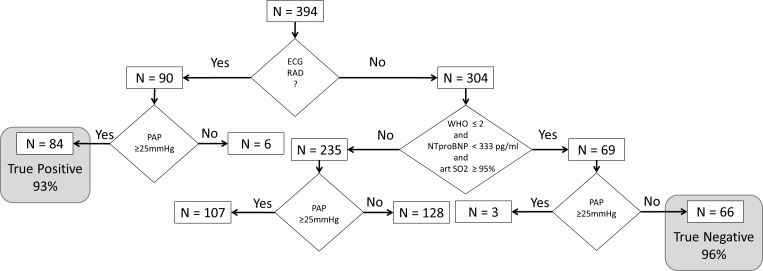
Algorithm to identify and to exclude pulmonary hypertension by simple non-invasive tools–data based on the analysis of the retrospective cohort. (RAD: right axis deviation, PAP: mean pulmonary arterial pressure, WHO: WHO functional class, NTproBNP: N-terminal pro brain natriuretic peptide, art SO2: arterial oxygen saturation).

**Fig 2:**
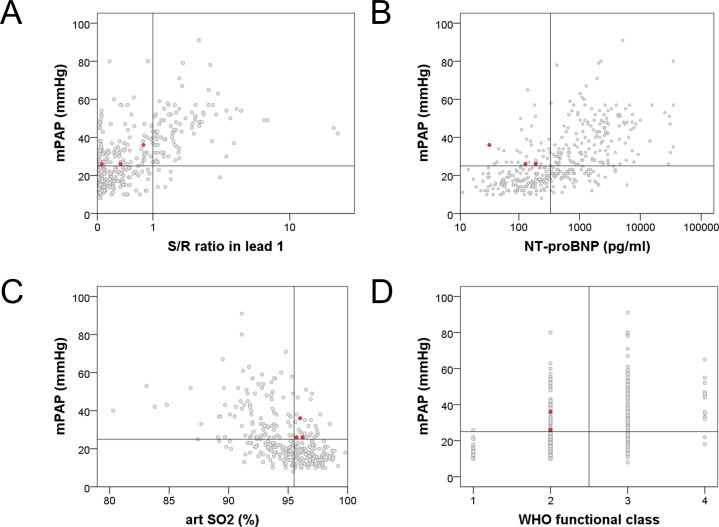
**A-D.** Associations between the simple non-invasive parameters and mean pulmonary arterial pressure–data based on the analysis of the retrospective cohort. The three red dots represent the patients with pulmonary hypertension who were missed by the algorithm. (mPAP: mean pulmonary arterial pressure, NTproBNP: N-terminal pro brain natriuretic peptide, art SO2: arterial oxygen saturation).

In the retrospective part n = 394 patients were included. 194/394 (49%) patients had PH confirmed by RHC (mPAP≥25mmHg). Patients’ characteristics are represented in Table 1. After performing the logistic regression, a two-step algorithm was developed (Fig 1, S1 Table): in step 1, the presence of RAD in the ECG was able to identify patients with a very high probability of PH, while in step 2, a low WHO functional class, high arterial SO_2_ and low NT-proBNP identified patients with a very low probability of PH. According to this model, in step 1, RAD was present in n = 90 patients. Out of these, PH was detected in n = 84 patients, and was missing in n = 6 subjects, revealing a positive predictive value of 93% (Fig 2A). Univariate all analyzed parameters were significant predictors for PH. Because of multicollinearity the following parameters were excluded: 6 Minute Walking distance, pO2, Borg dyspnea score at the end of the six minute walk test, FEV1 and FVC. In the multivariate analysis (Table 2) in step 2, NT-pro BNP, arterial SO_2_ and WHO functional class were the only remaining significant predictors for PH. The following parameters were also included in the multivariate analysis but did not reach significance: pCO2, DLCO and uric acid. Using cut off scores (NT-proBNP < 333pg/ml, Fig 2B; arterial SO_2_ ≥ 95.5% breathing room air, Fig 2C; WHO functional class I-II, Fig 2D) these three variables identified n = 69 out of the remaining 304 patients of which only n = 3 suffered from PH revealing a negative predictive value of 96%. Patients not fulfilling all of these three criteria had a significantly higher risk of PH (OR: 17.8, 95%CI 5.4–58.3). Combining both steps, the algorithm suggested a very high probability or very low probability of PH in 159/394 (40%) patients. Out of these 159 patients, the prediction was false positive in 6 patients (3.7%) and false negative in 3 patients (1.8%). Other combinations of potential predictors for PH did not result in a better prediction.

**Table 1:** Patients’ characteristics within the retrospective and prospective cohorts.

		retrospective (n = 394)			prospective (n = 168)		
		no PH (n = 200)	PH (n = 194)	p-value	no PH (n = 79)	PH (n = 89)	p-value
age (yr)		59.0 ± 14.4	64.5 ± 13.1	< .001	58.5 ± 13.3	64.6 ± 13.3	.003
Height (cm)		166.0 ± 7.5	167.3 ± 8.8	.116	168.5 ± 8.6	166.1 ± 12.8	.179
Weight (kg)		72.4 ± 15.0	75.1 ±16.7	.106	75.0 ± 14.6	79.3 ± 24.8	.180
BMI (kg/m^2^)		26.3 ± 5.1	26.8 ±5.7	.349	26.4 ± 4.4	29.6 ± 14.8	.058
mean PAP (mmHg)		17 (8–24)	38 (25–91)	< .001	16 (9–24)	40 (25–81)	< .001
PAWP (mmHg)		7.8 ± 3.2	10.5 ± 5.4	< .001	7.9 ± 3.3	11.9 ± 5.9	< .001
PVR (WU)		1.70 (0.59–4.43)	6.12 (1.40–25.25)	< .001	1.61 (0.27–4.51)	5.86 (1.70–23.72)	< .001
RAP (mmHg)		4.7 ± 3.1	7.8 ± 5.2	< .001	5.0 (0.0–18.0)	8.0 (2.0–23.0)	< .001
WHO functional class	I	27 (13.5%)	1 (0.5%)	< .001	11 (13.9%)	0 (0.0%)	< .001
	II	112 (56.0%)	58 (29.9%)		61 (77.2%)	27 (30.3%)	
	III	59 (29.5%)	123 (63.4%)		7 (8.9%)	57 (64.0%)	
	IV	2 (1.0%)	12 (6.2%)		0 (0.0%)	5 (5.6%)	
Sex	female	153(76.5%)	114 (58.8%)	< .001	55 (69.6%)	53 (59.6%)	.193
	male	47(23.5%)	80(41.2%)		24 (30.4%)	36 (40.4%)	
Collagen vascular disease	Yes	87 (43.5%)	13 (6.7%)	< .001	36 (45.6%)	8 (9.0%)	< .001
	No	113 (56.5%)	181 (93.3%)		43 (54.4%)	81 (91.0%)	
PH-Group	PAH		53 (27.3%)			28 (31.5%)	
	PH due to left heart disease		34 (17.5%)			13 (14.6%)	
	PH due to lung disease		52 (26.8%)			21 (23.6%)	
	CTEPH		38 (19.6%)			16 (18.0%)	
	PH with unclear / multifactorial mechanisms		17 (8.8%)			11 (12.4%)	
art SO2 (%)		95.7 ± 2.0	93.1 ± 3.1	< .001	96.1 ± 1.9	91.0 ± 6.4	< .001
art pO2 (%predicted)		73.9 ± 10.6	63.8 ± 10.2	< .001	74.1 ± 10.7	62.9 ±9.7	< .001
art pCO2 (% predicted)		36.2 ± 4.5	36.9 ± 7.4	.270	35.8 ± 4.0	37.2 ± 10.1	< .001
NT-proBNP (pg/ml)		188 (11–6038)	1248 (32–35000)	< .001	146 (16–2259)	1348 (41–35000)	< .001
6MWT (m)		410.9 ± 105.1	314.2 ± 128.2	< .001	449.8 ± 82.4	319.0 ± 123.5	< .001
R/S in I		0.06 (0.01–9.00)	0.80 (0.02–19.00)	< .001	0.06 (0.02–2.67)	0.73 (0.02–9.00)	< .001
FEV1 (%predicted)		84.8 ± 20.2	69.0 ± 22.3	< .001	90.9 ± 17.9	72.7 ± 23.1	< .001
FVC (%predicted)		86.2 ± 18.5	73.7 ± 19.4	< .001	98.7 ± 19.3	81.4 ± 23.5	< .001
DLCOcVA (%predicted)		84.6 ± 17.1	77.8 ± 26.0	.024	84.7 ± 18.5	73.7 ± 22.7	.001
DLCOcSB (%predicted)		77.1 ± 19.0	66.3 ± 23.2	< .001	79.3 ± 22.2	61.4 ± 19.1	< .001
HR (min^-1^)		72.4 ± 12.4	77.4 ± 15.0	< .001	70.1 ± 9.6	75.6 ± 17.2	.011
Uric acid (mg/dl)		5.7 ± 2.6	7.1 ± 2.4	< .001	5.4 ± 1.9	7.9 ± 5.6	< .001
Borg dyspnea scale at the end of 6MWT		2 (0–9)	4 (0–10)	< .001	2 (0–7)	3 (0–10)	< .001

Data are presented as means and standard deviation or median and interquartile range for continuous data and absolute and relative frequency for categorical data, respectively. BMI: body mass index, PAP: pulmonary arterial pressure, PAWP: pulmonary artery wedge pressure, PVR: pulmonary vascular resistance, RAP: right atrial pressure, PH: pulmonary hypertension, PAH: pulmonary arterial hypertension, CTEPH: chronic thromboembolic pulmonary hypertension, art SO2: arterial oxygen saturation, NT-proBNP: N terminal pro brain natriuretic peptide, 6MWD: six minute walk distance, FEV1: forced expiratory volume in the first second, FVC: forced vital capacity, DLCOcVA: diffusion capacity for carbon monoxide corrected for alveolar volume, DLCOcSB: single breath diffusion capacity for carbon monoxide, HR: heart rate, 6MWT: six minute walk test

**Table 2:** Risk for PH according to multivariate regression analysis.

		Odds ratio (95% Confidence interval)	p-value
WHO functional class	I, II	1	<0.001
	III, IV	3.7 (2.0–6.8)	
arterial SO_2_	≥ 95.5%	1	0.003
	< 95.5%	2.7 (1.4–5.1)	
NT-pro BNP	< 333pg/ml	1	<0.001
	≥ 333pg/ml	6.8 (3.7–12.5)	

NT-proBNP: N terminal pro brain natriuretic peptide; art SO_2_: arterial oxygen saturation

**Fig 3:**
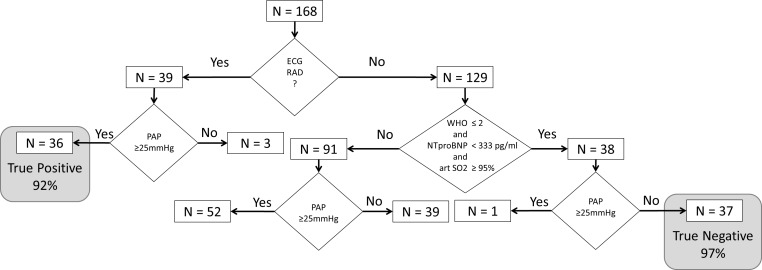
Algorithm to identify and to exclude pulmonary hypertension by simple non-invasive tools–data based on the analysis of the prospective cohort. (RAD: right axis deviation, PAP: mean pulmonary arterial pressure, WHO: WHO functional class, NTproBNP: N-terminal pro brain natriuretic peptide, art SO2: arterial oxygen saturation).

**Fig 4:**
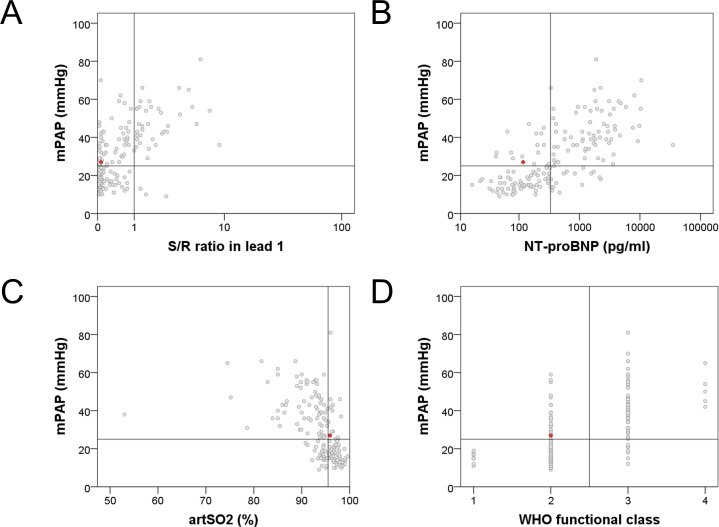
**A-D.** Associations between the simple non-invasive parameters and mean pulmonary arterial pressure–data based on the analysis of the prospective cohort. The red dot represents the patient with pulmonary hypertension who was missed by the algorithm. (mPAP: mean pulmonary arterial pressure, NTproBNP: N-terminal pro brain natriuretic peptide, art SO2: arterial oxygen saturation).

In the prospective part (Fig 3, S2 Table), n = 168 patients were included (see Table 1 for patients’ characteristics). 89/168 (53%) patients had PH. In step 1, RAD was present in n = 39 patients (Fig 4A). Within these, PH was detected in n = 36 patients, and was missing in n = 3 subjects, revealing a positive predictive value of 92%. In step 2, in the remaining n = 129 patients, the absence of PH was suggested in n = 38 patients (Fig 4B–4D), of which n = 1 suffered from PH revealing a negative predictive value of 97%. Combining both steps, the algorithm suggested either a very high or a very low probability in 77/168 (46%) patients. Out of these 77 patients, the prediction was false positive in 3 (3.9%) and false negative in 1 patient (1.3%).

**Fig 5:**
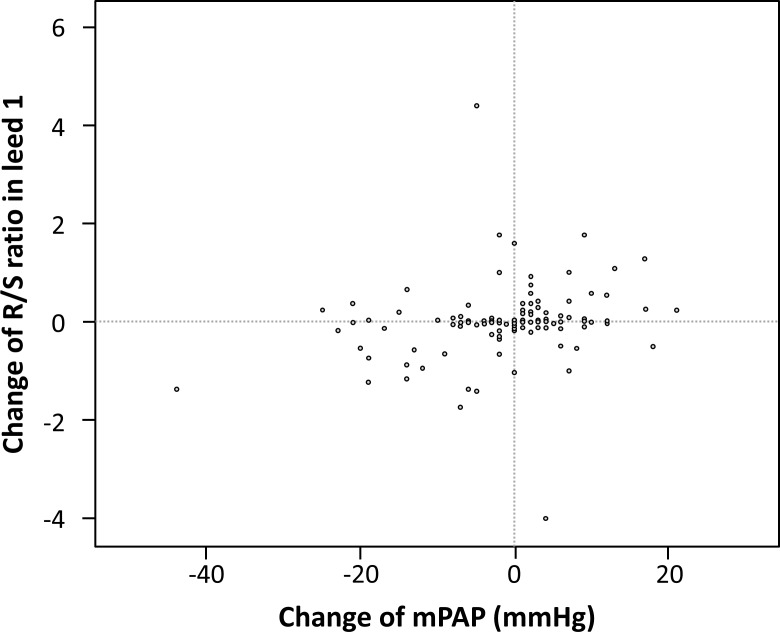
Association between changes in the electrical axis and changes in mean pulmonary arterial pressure in patients where two right heart catheterizations were performed at different time points. (mPAP: mean pulmonary arterial pressure).

During the time frame of the study there were n = 151 patients with at least two RHC investigations and corresponding non-invasive measures. In these patients, the change of the ECG electrical axis showed a moderate correlation with the change in mPAP (ρ = 0.28, p = 0.001, Fig 5). A similar correlation was found between changes in mPAP and NT-proBNP (ρ = 0.22, p = 0.007, not shown). No significant correlations were found between changes in mPAP and changes in WHO functional class or arterial SO_2_.

## Discussion

In this study we aimed to develop an algorithm based on simple, non-invasive, easily reproducible, and widely available parameters in order to predict or exclude manifest pulmonary hypertension in patients at risk for PH. The investigated variables included the presence of RAD in ECG, blood gas analysis, pulmonary function tests, NT-proBNP, uric acid, six-minute walk distance, Borg dyspnea score at the end of the six-minute walk test and WHO functional class. All of these simple variables are assessed during the diagnostic work-up of PH according to current guidelines. Several of the above parameters [11–14] are considered to have prognostic relevance in PH, however, their diagnostic relevance has not yet been systematically addressed. More complex examinations that rely on the readers’ skills and experience such as echocardiography, were deliberately not included. The algorithm was developed based on a retrospective analysis of a large patient population undergoing right heart catheterization and was then validated in a prospective manner. The validation cohort did not only confirm the result of the retrospective study but showed even a little higher negative predictive value. The suggested algorithm including the analysis of right axis deviation in the ECG, the level of NT-proBNP, arterial SO_2_ and WHO functional class may be used at an early stage of the diagnostic decision-tree to identify patients with very high and very low likelihood for PH.

### ECG–right axis deviation

In recent years a relevant role for ECG has been suggested in screening algorithms distinguishing pre- and post-capillary PH [15] and identifying systemic sclerosis associated PH [6]. In this latter study, RAD (electrical axis of the heart ≥90°) was present in 13% of systemic sclerosis patients with pulmonary arterial hypertension (PAH), but only in 3% of patients without PH. In a large echocardiography study performed on 372 patients (n = 282 with PH and n = 90 without PH) RAD (defined as electrical axis of the heart ≥110°) was associated with a high positive predictive value (89%) but low negative predictive value (26%) for PH [16]. In our patient population, 23% had a RAD and this was associated with a high positive predictive value (92–93%) and a modest negative predictive value (59–65%) for manifest PH as diagnosed by right heart catheterization. This corresponds to a previous study [17], although in that study the positive predictive value could not be calculated due to the study design. Therefore, our data confirm that RAD may be strongly suggestive for the presence of PH, but its absence does not exclude PH (Figs 2A and 4A).

In previous studies also other ECG parameters such as P wave amplitude, P wave axis, signs of right and left ventricular strain etc. were analyzed [15]. In our study we deliberately used only one single robust and very simple ECG parameter, which appeared to be relevant based on the earlier studies and allows automatic interpretation.

We also looked for possible confounders in our analysis, which may have influenced the electrical axis of the heart. Neither age (p = 0.786) nor pulmonary arterial wedge pressure (PAWP) as a variable for left heart disease (p = 0.667) was significantly associated with the electrical axis of the heart. Systemic blood pressure was weakly negatively associated with RAD (ρ = -0.279, p<0.001) and males presented more often with RAD than females in the retrospective (p = 0.001) but not in the prospective cohort (p = 0.222).

Recent studies suggested that ECG may indicate disease progression [18] and therapy response [19] in PAH. We also found a modest correlation between the change in the electrical axis of the heart and mPAP between baseline and follow-up examinations. Although this correlation was comparable to the correlation between mPAP and NT-proBNP changes, the clinical relevance of such findings remains low.

### NT-proBNP

Brain natriuretic peptide (BNP) and NT-proBNP are established biomarkers with prognostic relevance in PAH [20–22], although they may depend on age and renal function [23]. Besides being recommended as a marker of clinical progression, NT-proBNP has been suggested to be included in screening algorithms for PH. In different clinical settings, various NT-proBNP thresholds may be clinically relevant. In the DETECT study [6], a continuous scale, but no single threshold for NT-proBNP was used, while another recent -PAH screening algorithm in systemic sclerosis recommended a threshold of 210 pg/ml [24]. In the study of Bonderman et al., a low NT-proBNP threshold (80pg/ml) distinguished pre- and post-capillary PH [15]. In our algorithm, as part of a combination of parameters, the optimal threshold was found to be in a higher range (333pg/ml).

### WHO functional class and arterial SO_2_

The most frequently used, easy and well established method to describe the physical limitation in patients with PH is the WHO functional class. Although it is very subjective, and the stratification strategy may vary among centers and individual physicians, the WHO functional class performs surprisingly well in clinical studies as secondary end-point and represents one of the most important prognostic parameters in PAH [25]. The measurement of oxygen saturation is simple and can be taken from arterial blood gas analysis or non-invasively from pulse oximetry. Both WHO functional class and arterial SO_2_ are unspecific for pulmonary hypertension [26]. However, in combination with our other parameters they proved to be very useful to identify patients with no PH.

### Clinical relevance of our findings

The group of patients with very high probability for PH were those with RAD (electrical axis of the heart >90°) in the ECG, revealing a positive predictive value of 92–93%. This may suggest that in dyspnea patients or a disease associated with PAH, a right axis deviation in the ECG is highly suspicious for PH and further clinical examinations (echocardiography, eventually right heart catheterization) are strongly recommended.

Patients with very low probability for PH were characterized by any ECG axis other than RAD, low NT-proBNP, good oxygen saturation and low WHO functional class. Our results may thus suggest a reasonable role for these parameters in an active decision against a diagnostic work-up for PH and support the concept of clinical and laboratory patterns facilitating specific diagnostic decisions [6,15,27–29]. Of course, such criteria cannot replace specific methods like echocardiography and right heart catheterization. On the other hand, in many clinical situations we discuss diagnostic procedures with patients at moderate risk for PH and both the physician and the patient may profit from prediction rules to guide the shared decision making. Therefore, we believe that our results may be useful for guiding the decisions towards specific diagnostics in individual patients at risk for pulmonary hypertension.

### Limitations

As the most important limitation of our study, our patient collective was typical for a PH center but may be different in primary care settings or specialized heart or lung clinics. All patients had either unexplained dyspnea or an established risk factor for PH and the findings (including reported PPV and NPV) may not be valid in patients without these characteristics or in the general population. In addition, we have to accept that besides correctly predicting PH or “no PH” in about half of the patients, our algorithm was not able to provide additional help to indicate the presence or absence of pulmonary hypertension in the other half.

## Conclusion

Our suggested 2-step algorithm recognizes patients with either a very high or a very low probability for pulmonary hypertension in nearly half of the patients at risk for PH. This result can be achieved by the systematic use of four simple non-invasive parameters: right axis deviation in ECG, SO_2_, NT-proBNP and WHO functional class.

## Supporting Information

S1 Table Individual data of patients assessed in the retrospective part of the study. (SAV)

S2 Table Individual data of patients assessed in the prospective part of the study. (SAV)

## References

[1] Galie N, Humbert M, Vachiery JL, Gibbs S, Lang I, Torbicki A, et al 2015 ESC/ERS Guidelines for the diagnosis and treatment of pulmonary hypertension: The Joint Task Force for the Diagnosis and Treatment of Pulmonary Hypertension of the European Society of Cardiology (ESC) and the European Respiratory Society (ERS): Endorsed by: Association for European Paediatric and Congenital Cardiology (AEPC), International Society for Heart and Lung Transplantation (ISHLT). Eur Heart J. 2016;37: 67–119. 10.1093/eurheartj/ehv317 26320113

[2] Abramson SV, Burke JF, Kelly JJ Jr, Kitchen JG 3rd, Dougherty MJ, Yih DF, et al Pulmonary hypertension predicts mortality and morbidity in patients with dilated cardiomyopathy. Ann Intern Med. 1992;116: 888–895. 1580444 10.7326/0003-4819-116-11-888

[3] Lam CS, Borlaug BA, Kane GC, Enders FT, Rodeheffer RJ, Redfield MM. Age-associated increases in pulmonary artery systolic pressure in the general population. 2009;119: 2663–70. 10.1161/CIRCULATIONAHA.108.838698 PMC2753443 19433755

[4] Oswald-Mammosser M, Weitzenblum E, Quoix E, Moser G, Chaouat A, Charpentier C, et al Prognostic factors in COPD patients receiving long-term oxygen therapy. Importance of pulmonary artery pressure. 1995;107: 1193–8. 7750305 10.1378/chest.107.5.1193

[5] Fisher MR, Forfia PR, Chamera E, Housten-Harris T, Champion HC, Girgis RE, et al Accuracy of Doppler echocardiography in the hemodynamic assessment of pulmonary hypertension. 2009;179: 615–21. 10.1164/rccm.200811-1691OC 19164700 PMC2720125

[6] Coghlan JG, Denton CP, Grunig E, Bonderman D, Distler O, Khanna D, et al Evidence-based detection of pulmonary arterial hypertension in systemic sclerosis: the DETECT study. Ann Rheum Dis. 2014;73: 1340–1349. 10.1136/annrheumdis-2013-203301 23687283 PMC4078756

[7] Galie N, Hoeper MM, Humbert M, Torbicki A, Vachiery JL, Barbera JA, et al Guidelines for the diagnosis and treatment of pulmonary hypertension: The Task Force for the Diagnosis and Treatment of Pulmonary Hypertension of the European Society of Cardiology (ESC) and the European Respiratory Society (ERS), endorsed by the International Society of Heart and Lung Transplantation (ISHLT). 2009;30: 2493–537. 10.1093/eurheartj/ehp297 19713419

[8] Galie N, Torbicki A, Barst R, Dartevelle P, Haworth S, Higenbottam T, et al Guidelines on diagnosis and treatment of pulmonary arterial hypertension. The Task Force on Diagnosis and Treatment of Pulmonary Arterial Hypertension of the European Society of Cardiology. 2004;25: 2243–78. 10.1016/j.ehj.2004.09.014 15589643

[9] ATS Committee on Proficiency Standards for Clinical Pulmonary Function Laboratories. ATS statement: guidelines for the six-minute walk test. Am J Respir Crit Care Med. 2002;166: 111–117. 10.1164/ajrccm.166.1.at1102 12091180

[10] Kovacs G, Olschewski H. Cardiac Catheterization In: Peacock AJ, Naeije R, Rubin LJ, editors. Pulmonary Circulation.: CRC Press Taylor & Francis Group; 2016 pp. 186–196.

[11] Olsson KM, Sommer L, Fuge J, Welte T, Hoeper MM. Capillary pCO2 helps distinguishing idiopathic pulmonary arterial hypertension from pulmonary hypertension due to heart failure with preserved ejection fraction. Respir Res. 2015;16: 34-015-0194-6. 10.1186/s12931-015-0194-6 PMC4358848 25853979

[12] Hoeper MM, Pletz MW, Golpon H, Welte T. Prognostic value of blood gas analyses in patients with idiopathic pulmonary arterial hypertension. Eur Respir J. 2007;29: 944–950. 10.1183/09031936.00134506 17301100

[13] Foris V, Kovacs G, Tscherner M, Olschewski A, Olschewski H. Biomarkers in pulmonary hypertension: what do we know? Chest. 2013;144: 274–283. 10.1378/chest.12-1246 23880678

[14] Miyamoto S, Nagaya N, Satoh T, Kyotani S, Sakamaki F, Fujita M, et al Clinical correlates and prognostic significance of six-minute walk test in patients with primary pulmonary hypertension. Comparison with cardiopulmonary exercise testing. 2000;161: 487–92. 10.1164/ajrccm.161.2.9906015 10673190

[15] Bonderman D, Wexberg P, Martischnig AM, Heinzl H, Lang MB, Sadushi R, et al A noninvasive algorithm to exclude pre-capillary pulmonary hypertension. Eur Respir J. 2011;37: 1096–1103. 10.1183/09031936.00089610 20693249

[16] Al-Naamani K, Hijal T, Nguyen V, Andrew S, Nguyen T, Huynh T. Predictive values of the electrocardiogram in diagnosing pulmonary hypertension. Int J Cardiol. 2008;127: 214–218. 10.1016/j.ijcard.2007.06.005 17651847

[17] Rich S, Dantzker DR, Ayres SM, Bergofsky EH, Brundage BH, Detre KM, et al Primary pulmonary hypertension. A national prospective study. Ann Intern Med. 1987;107: 216–223. 3605900 10.7326/0003-4819-107-2-216

[18] Tonelli AR, Baumgartner M, Alkukhun L, Minai OA, Dweik RA. Electrocardiography at diagnosis and close to the time of death in pulmonary arterial hypertension. Ann Noninvasive Electrocardiol. 2014;19: 258–265. 10.1111/anec.12125 24372670 PMC4004655

[19] Henkens IR, Gan CT, van Wolferen SA, Hew M, Boonstra A, Twisk JW, et al ECG monitoring of treatment response in pulmonary arterial hypertension patients. 2008;134: 1250–7. 10.1378/chest.08-0461 18641107

[20] Leuchte HH, Holzapfel M, Baumgartner RA, Ding I, Neurohr C, Vogeser M, et al Clinical significance of brain natriuretic peptide in primary pulmonary hypertension. J Am Coll Cardiol. 2004;43: 764–770. 10.1016/j.jacc.2003.09.051 14998614

[21] Leuchte HH, Holzapfel M, Baumgartner RA, Neurohr C, Vogeser M, Behr J. Characterization of brain natriuretic peptide in long-term follow-up of pulmonary arterial hypertension. Chest. 2005;128: 2368–2374. 10.1378/chest.128.4.2368 16236896

[22] Fritz JS, Blair C, Oudiz RJ, Dufton C, Olschewski H, Despain D, et al Baseline and follow-up 6-min walk distance and brain natriuretic peptide predict 2-year mortality in pulmonary arterial hypertension. Chest. 2013;143: 315–323. 10.1378/chest.12-0270 22814814 PMC4694187

[23] Leuchte HH, El Nounou M, Tuerpe JC, Hartmann B, Baumgartner RA, Vogeser M, et al N-terminal pro-brain natriuretic peptide and renal insufficiency as predictors of mortality in pulmonary hypertension. Chest. 2007;131: 402–409. 10.1378/chest.06-1758 17296640

[24] Thakkar V, Stevens W, Prior D, Youssef P, Liew D, Gabbay E, et al The inclusion of N-terminal pro-brain natriuretic peptide in a sensitive screening strategy for systemic sclerosis-related pulmonary arterial hypertension: a cohort study. Arthritis Res Ther. 2013;15: R193 10.1186/ar4383 24246100 PMC3978999

[25] Sitbon O, Humbert M, Nunes H, Parent F, Garcia G, Herve P, et al Long-term intravenous epoprostenol infusion in primary pulmonary hypertension: prognostic factors and survival. 2002;40: 780–8. 12204511 10.1016/s0735-1097(02)02012-0

[26] Galie N, Rubin L, Hoeper M, Jansa P, Al-Hiti H, Meyer G, et al Treatment of patients with mildly symptomatic pulmonary arterial hypertension with bosentan (EARLY study): a double-blind, randomised controlled trial. 2008;371: 2093–100. 10.1016/S0140-6736(08)60919-8 18572079

[27] Benza RL, Gomberg-Maitland M, Miller DP, Frost A, Frantz RP, Foreman AJ, et al The REVEAL Registry risk score calculator in patients newly diagnosed with pulmonary arterial hypertension. Chest. 2012;141: 354–362. 10.1378/chest.11-0676 21680644

[28] Nickel N, Golpon H, Greer M, Knudsen L, Olsson K, Westerkamp V, et al The prognostic impact of follow-up assessments in patients with idiopathic pulmonary arterial hypertension. Eur Respir J. 2012;39: 589–596. 10.1183/09031936.00092311 21885392

[29] Kane GC, Maradit-Kremers H, Slusser JP, Scott CG, Frantz RP, McGoon MD. Integration of clinical and hemodynamic parameters in the prediction of long-term survival in patients with pulmonary arterial hypertension. Chest. 2011;139: 1285–1293. 10.1378/chest.10-1293 21071530

